# Use of Closed Incision Negative Pressure Therapy for Massive Subcutaneous Emphysema

**DOI:** 10.7759/cureus.7399

**Published:** 2020-03-24

**Authors:** Benjamin C Taylor, Sean McGowan

**Affiliations:** 1 Orthopaedic Surgery, Grant Medical Center, Columbus, USA

**Keywords:** flail chest, negative pressure wound therapy, wound vac, subcutaneous emphysema

## Abstract

Subcutaneous emphysema is typically due to an air leak through the parietal pleura, allowing air to escape from the lung parenchyma into the adjacent soft tissue. Most cases are benign and self-limiting; however, when enough air is forced into the subcutaneous tissues allowing the air to spread into the neck, tracheal compression and respiratory distress can occur. Tube thoracotomy and endotracheal intubation are generally sufficient to overcome this respiratory compromise. However, occasionally other invasive measures are required to allow the air leak to resolve. Traditionally, this would involve placement of an incision or two into the anterior chest wall to allow decompression to the outside environment. Limited evidence exists regarding negative pressure wound therapy devices being used successfully with open incisions for the management of massive subcutaneous emphysema.

We present the initial case of successful use of a loosely closed incision negative pressure therapy for massive subcutaneous emphysema. In this instance, the patient's thoracic injury was successfully stabilized and use of the negative pressure therapy device allowed the incisions to be closed with a much more cosmetically pleasing result.

## Introduction

Subcutaneous emphysema is typically caused by any ongoing air leak that can divide and diffuse into and between the fascial planes. Treatment of massive subcutaneous emphysema usually consists of supportive care and tube thoracostomy placement, allowing for reapproximation of the parietal and visceral pleura and halting the escape of air into the subcutaneous tissues. Occasionally in cases of high flow air leaks, surgery may be even required to halt the escape of air from the pleural cavity.

Less invasive techniques have been described, including “blowhole” incisions and percutaneous angiocatheter placement [1,2]. Minimally invasive techniques such as these would be preferable in a multiply injured patient to avoid additional systemic burden. Negative pressure wound therapy devices have been used in many different aspects of patient care with positive results, and there are several case reports of successful negative pressure therapy for the treatment of massive subcutaneous emphysema [3-5]. Use of negative pressure therapy for this condition carries the benefit of allowing continuous high-level suction to create an efficient mechanism for trapped air removal, prevention of clotting or blockage that can be associated with closed suction drainage or catheter systems, and creation of a quick, sterile mechanism for subcutaneous emphysema resolution. This is especially important when more invasive thoracic surgical intervention is too demanding on the patient’s current condition. Closed incision negative pressure wound therapy (ciNPWT) has been shown to be effective in decreasing wound complications, especially in high-risk patients, and allows for stabilization of the wound edges as well as the removal of exudate [6]. However, the use of ciNPWT has not been well described in the use of loosely closed incisions; this theoretically would carry the benefits of allowing wound edge stabilization and removal of exudate and drainage, as with fully closed incisions, but would also allow for continued drainage of air in cases of massive subcutaneous emphysema such as this particular case.

## Case presentation

**Figure 1:**
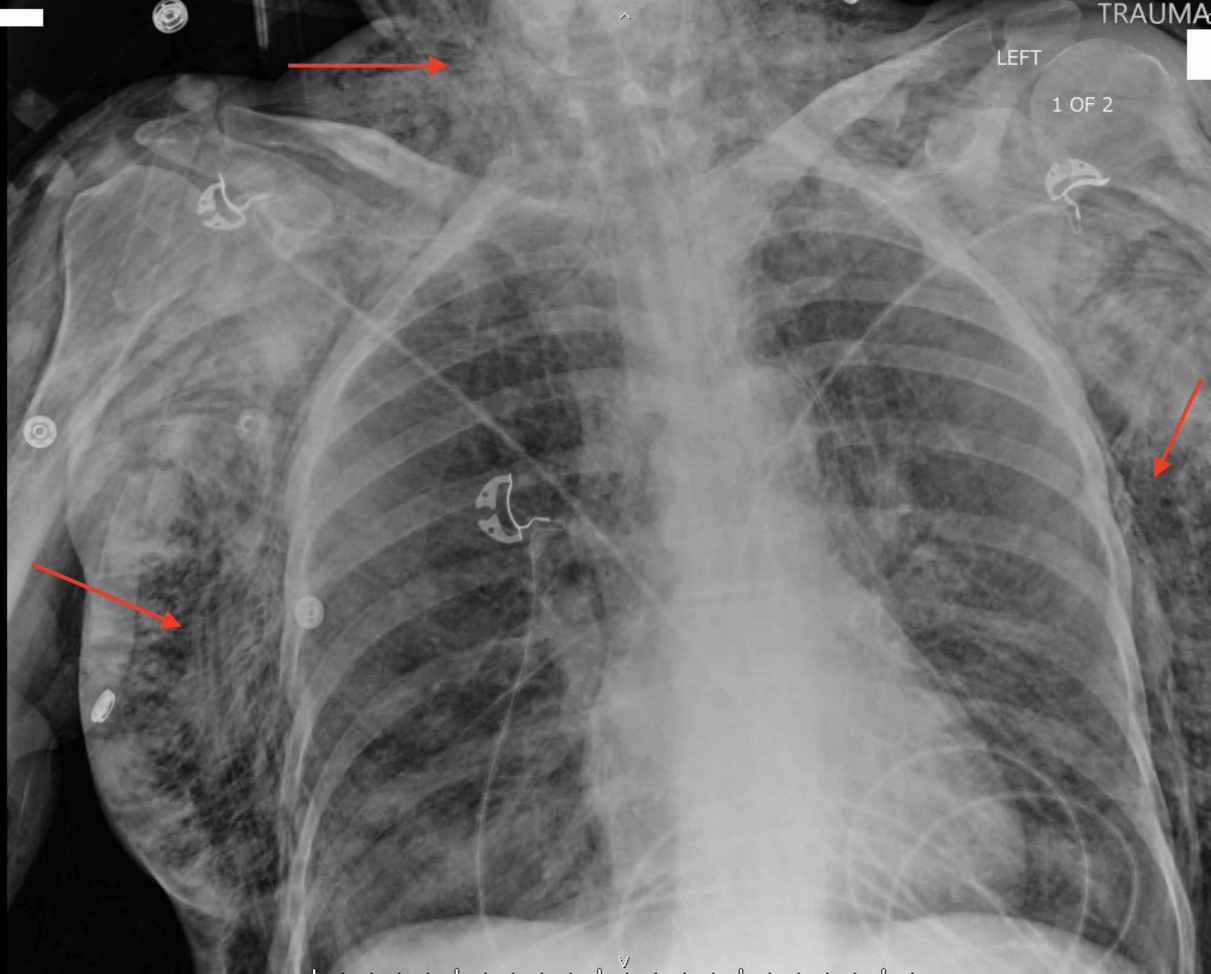
Massive Subcutaneous Emphysema Initial trauma chest radiograph showing significant widespread subcutaneous air (arrows pointing to areas with large amounts of subcutaneous emphysema).

A 52-year-old female sustained an unwitnessed fall down approximately fifteen stairs at her home due to loss of balance while being intoxicated. She did have a medical history significant for bipolar disorder, cardiomyopathy, and chronic obstructive pulmonary disease requiring the use of two liters of oxygen via nasal cannula nightly. Initial evaluation in the trauma bay revealed the patient to be tachypneic and agitated, with a significant amount of subcutaneous crepitance to her chest, shoulders, neck and even into her face. Full evaluation of the patient revealed right fourth through seventh rib fractures, bilateral pneumothoraces, pneumomediastinum, massive subcutaneous emphysema, and an anion gap metabolic acidosis (Figure 1). 

She was treated initially with bilateral tube thoracostomy and admitted to the intensive care unit for monitoring, pulmonary toilet, and correction of her acidosis. Initially, her pneumothoraces appeared to have resolved, however, by 48 hours post-admission, she had developed a right-sided apical pneumothorax and began experiencing increasing respiratory distress. Unfortunately, on the third hospital day, she underwent fiberoptic-assisted intubation by anesthesia staff as the result of tracheal compression from massive subcutaneous emphysema. The patient also had begun to require vasopressor support. She remained relatively stable after intubation, and the team was able to discontinue her vasopressor support, but continued to show evidence of pneumomediastinum and was unable to wean from the ventilator due to continued tracheal compression from the massive subcutaneous emphysema. 

**Figure 2:**
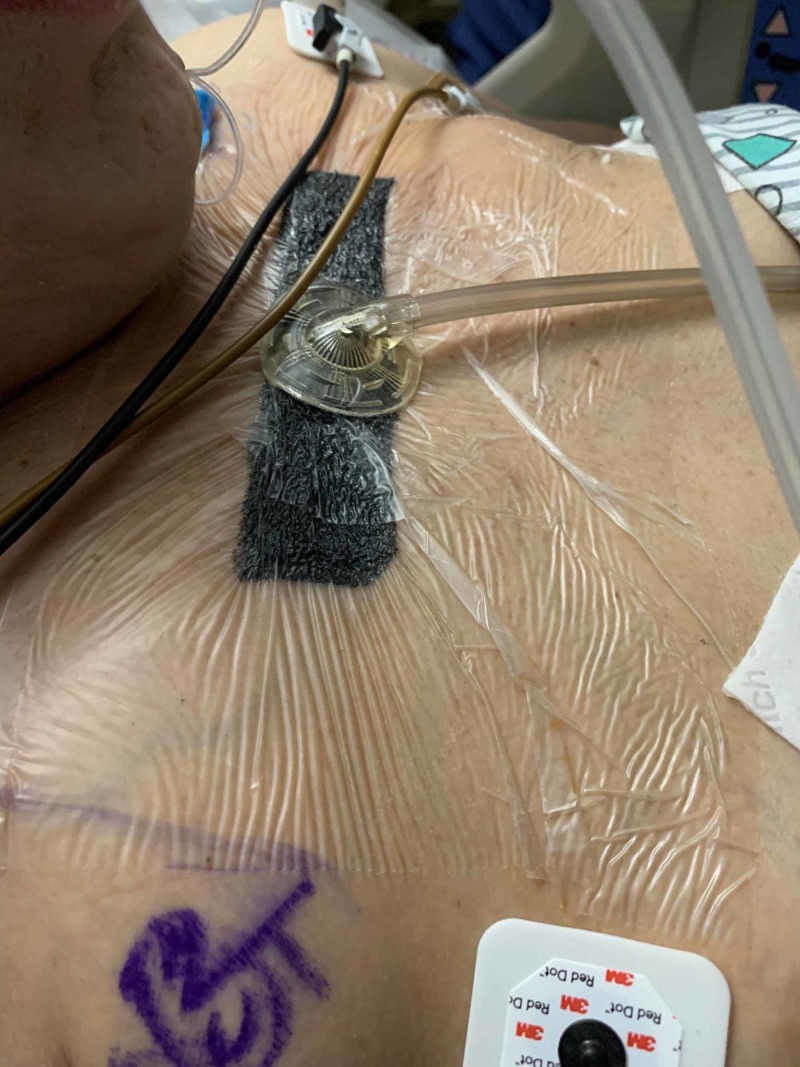
Clinical Photo Photo of the anterior thoracic closed incision negative pressure wound therapy (ciNPWT) covering the loosely approximated "blow hole" incisions.

On the sixth day post-injury, a chest wall venting “blowhole” procedure was performed by making bilateral three-centimeter incisions through the skin and subcutaneous tissue in a transverse fashion inferior to the medial clavicles. Dissection through the fascia revealed an immediate rush of air bilaterally. As the air leak was significant, each of the incisions were loosely closed in a simple fashion and covered with a single piece of black foam with Adaptic underneath each to allow continued air escape through the two incisions. A bridging piece of foam was utilized over a single piece of protective film between the incisions, and ciNPWT was used with a setting of -125mmHg continuous pressure (Figure 2).

**Figure 3:**
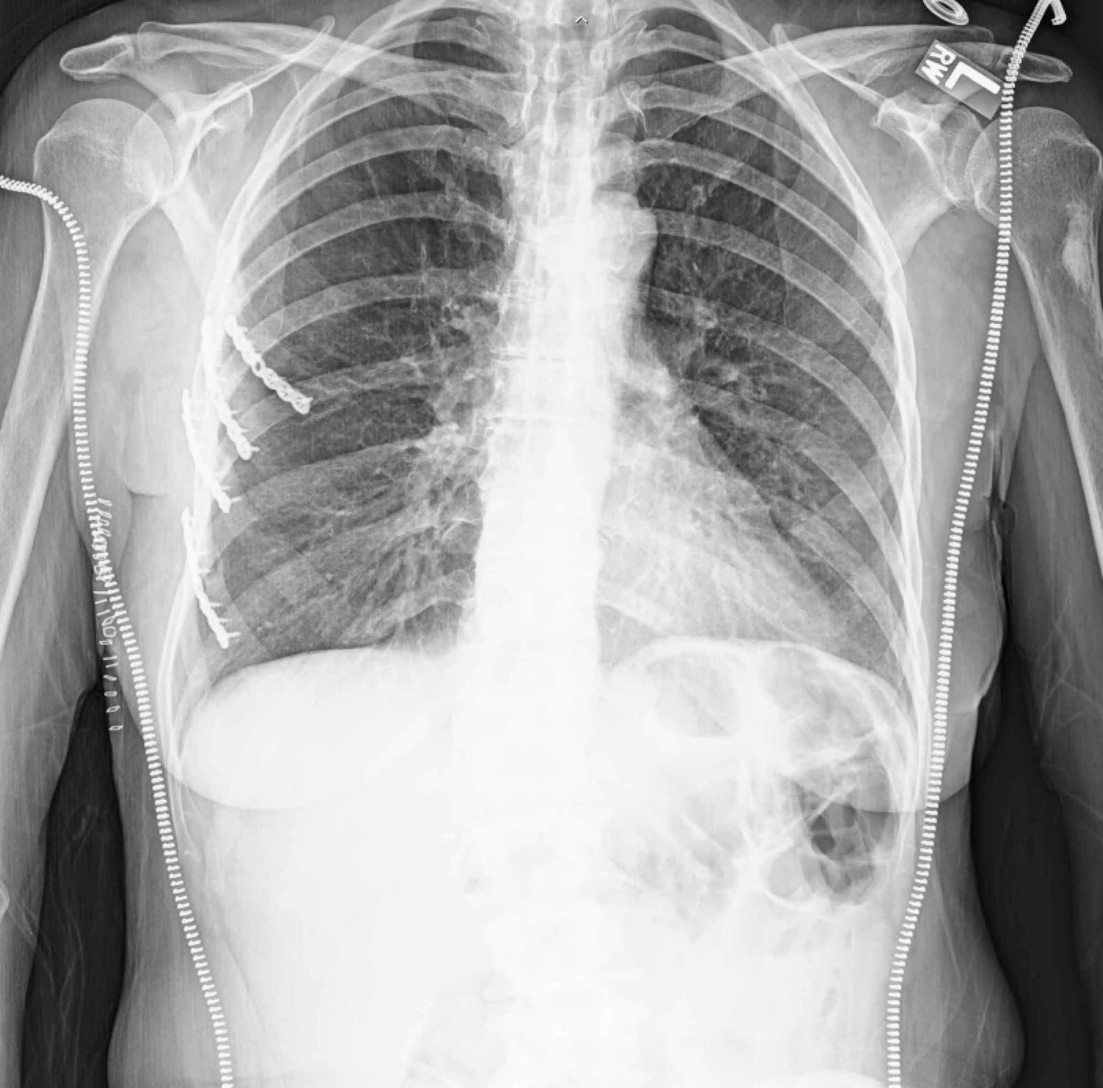
Final Radiograph Fully resolved pneumothorax and subcutaneous emphysema; rib fractures have also gone onto successful union.

The following day, she underwent surgical fixation of the chest wall with instrumentation of ribs four through seven, and also underwent primary repair of some of the intercostal muscle injury. Postoperatively, a small apical pneumothorax was still noted, but she continued to show signs of improvement. The subcutaneous air continued to decrease to a point that she was extubated two days after her rib fracture surgery (post-injury day nine). On post-injury day fourteen, the left thoracostomy tube and sponges were removed. She subsequently went home the following day after staying overnight for observation following removal. Most recent follow up in the outpatient office revealed two well-healed incisions about the anterior chest, as well as united rib fractures and full resolution of her subcutaneous emphysema (Figure 3). 

## Discussion

Subcutaneous emphysema can be caused by an ongoing air leak that divides and diffuses in between fascial planes; treatment of this condition is usually with supportive care or thoracotomy tube placement to allow reapproximation of the parietal and visceral pleura and halting the escape of air into the subcutaneous tissues. In instances of recalcitrant disease, formal drainage is necessary with either a single or multiple catheters or decompressive open incisions [1,2]. The first description of negative pressure therapy for massive subcutaneous emphysema was in 2009, where the sponges were used after incisions were placed down to and through fascia after persistent subcutaneous air pressure leading to respiratory failure and high levels of positive end expiratory pressure needed to maintain ventilation [7]. After this initial description, several other small case series were presented, showing successful eradication of subcutaneous emphysema with the use of multiple open decompressing incisions and placing foam sponges into the incisions; indications for the technique included pneumomediastinum-induced cardiac tamponade and airway obstruction [7-9]. Interestingly, these techniques utilized varying suction pressures ranging from -100 to -150mmHg, with several notes of wound and dressing related pain at the higher suction levels. The sponges in these cases were tunneled under the skin and fascia, and note is made of the risk of bleeding, pain, and wound healing complications with the tunneling required with this particular technique. 

ciNPWT has been shown to improve outcomes with healing of closed incisions via improvements in skin edge tensile forces, wound environment stabilization, edema control, improved removal of exudate, prolonged availability of a sterile dressing, and increased blood and lymphatic flow [3,6]. The advantages of ciNPWT were also evident in this case, as the continuous suction was able to be applied through a sterile dressing and a set of loosely approximated incisions, leading to successful resolution of a massive subcutaneous emphysema. A fully watertight closure may or may not have allowed successful egress for the air leak, and it was decided to loosely approximate the edges of the wounds as in instances where continued fluid drainage would be desired. By using ciNPWT and sponges outside of the patient, this allowed avoidance of potential pain and bleeding associated with sponge changes as well as prevented collapse of the sponge underneath a tunneled wound. Although this report shows that ciNPWT is a viable technique for eradication of subcutaneous emphysema, comparison of sponge use superficially or internally would be valid and could be performed either in a clinical setting or with use of cadaver or a representative model. Additionally, investigation of the influence of differing suction pressures and intermittent suction settings would be useful in better characterizing this technique.

## Conclusions

ciNPWT can successfully be utilized in instances of massive subcutaneous emphysema. This procedure can be performed either at bedside in emergent conditions or more formally in an operating room setting. Further analysis of this topic would include pressure settings, intermittent versus continuous pressure settings, use of unilateral versus bilateral incisions, and comparison of sponge use internal or external to the body. 
